# A Test Case and Its Issues

**Published:** 1913-03-29

**Authors:** 


### NATIONAL INSURANCE ACT DEPARTMENT.

### XLVIII.—A Test Case and its Issues.

The case—*Heard* v. *Pickthorne and others*—which came before Mr. Justice Bailhache in the King's Bench Division on March 18 is interesting and important, not so much on the merits of the case itself, but in regard to the larger issues which are involved. The matter actually in dispute was, indeed, singularly trifling, the claim being for no more than 5s. But, as so often happens when legal proceedings are taken and the sum in dispute is small, important principles were involved of much wider significance than those attaching to the particular case. The case was, in fact, in the nature of a test case, and the judgment, having been placed on record, will, doubtless, be followed as a precedent.

Before dealing with the larger issues it is necessary for us to give a brief recital of the facts, and to state the result of the action.

### The Facts of the Case.

The plaintiff had been for many years a member of the Abbey Court Branch of the Ancient Order of Foresters Friendly Society, and when the National Insurance Act came into operation he continued as a voluntary member, and also joined the Approved Society, which the Foresters had formed, for the purpose of receiving the State benefits. In January last he fell ill, and declared on the funds for sickness benefit. His claim on the voluntary section was admitted and paid, but the State benefit was declined on the ground that the medical certificate which was produced in support of his claim was not signed by a *panel* doctor. It appeared that the committee of management of the Society had passed a resolution that all claims for benefit were to be accompanied by a medical certificate furnished by a panel doctor. The plaintiff declined to produce such a certificate, holding that the medical certificate which he had produced, and which had been accepted by the Society on its voluntary side as sufficient evidence of his incapacity for work was also sufficient evidence to entitle him to claim under the National Insurance Act. Having failed to arrive at an agreement with the Society in the matter the action was brought against the trustees to have the resolution requiring a panel doctor's certificate as evidence of incapacity to be set aside as illegal, *ultra vires*, and unenforceable.

The point at issue, therefore, was the validity of a resolution of the Society. In support of the action it was claimed that there was nothing in the Act nor in the rules of the Society which would require the production of a certificate from a panel doctor.

The plaintiff's case was a strong one, and on its merits would, doubtless, have been decided in his favour, but, unfortunately, the defence was set up that the Court had no jurisdiction in the matter. The ground of this defence was that under Section 67 of the Act any dispute between a member and his society is required to be settled according to the rules of the society, with the right of appeal to the Insurance Commissioners, whose decision is to be final and conclusive. The rules provided for arbitration, and, although it was argued on behalf of the plaintiff that the refusal to accept a medical certificate other than that of a practitioner on the panel was so far beyond the powers of the Society as to allow the Court to interfere, Mr. Justice Bailhache decided that the plaintiff's remedy was by means of arbitration with the right of appeal to the Insurance Commissioners. The application was, therefore, dismissed, with costs. So much for the immediate facts of the case and its result.

### Medical Certificates and Sickness Benefit.

The first of the larger issues that it involves is the question of the acceptance of certificates by approved societies other than those of panel doctors. During the first few weeks after the benefits became payable there was considerable doubt among approved societies generally whether they were justified in accepting certificates except those furnished by panel doctors. This was probably the case with the Foresters, and it was most likely for this reason that the resolution was passed. Other societies are known to have done the same thing. The doubts arose because the societies are administering State funds, and have to satisfy the Insurance Commissioners that the claims are properly incurred, and the payments have to be duly vouched. Unless the claim is in order in all respects the society is liable to be surcharged. Hence the need for caution; and knowing the attitude of the Commissioners in regard to the panel system, the society officials determined to err on the safe side. It may have been, too, that the societies wished to bring before their members the full advantages of the Act, and to point out that they could obtain certificates without charge by applying to a panel doctor. The question had not been raised until the benefits had actually become payable, and action had to be taken without waiting to consult the Insurance Commissioners. There were, therefore, good grounds for the attitude adopted by the societies. We understand, however, that when the matter was submitted to the Insurance Commissioners it was decided that it is not essential that claims should be supported by certificates from panel doctors. Sickness benefit is now, therefore, being paid by most approved societies provided a medical certificate is produced, whether signed by a doctor who is on the panel or not.

In these circumstances it seems to be clear that, although the case to which we referred was dismissed in the King's Bench Division, the plaintiff would succeed, if not in the arbitration proceedings, at least on appeal to the Insurance Commissioners. To this extent the position is satisfactory, but there is another matter that demands attention.

### The Validity of Rules.

It is recognised that in all societies, of whatever description, there must be rules, and it is supposed that on joining a society the member becomes acquainted with those rules and agrees to be bound by them. There are, however, certain circumstances in which it is possible for the rules to be called in question in spite of their apparent legal authority. The famous Osborne judgment is a case in point. In the particular case with which we have been dealing it was not one of the formal rules of the Society which was in dispute, but a resolution of the Society—presumably that of the committee of management—and in view of the provisions of the rules it should have been apparent that an action in the High Court was not the method to be adopted in order to obtain redress. There are, however, certain rules of the approved societies which have obtained the sanction of the Insurance Commissioners, but which may in course of time be called in question. As an instance we may mention that several societies of which we have definite knowledge have a rule regulating the conduct of members whilst in receipt of sick benefit, and prescribing that the member shall not leave the parish in which he is resident without the consent of the committee of management. This rule follows closely the usual practice of Friendly Societies, and it is probable that those who formerly belonged to such societies would be perfectly familiar with the rule.

### One That May Cause Trouble.

Two cases, however, recently came to our notice within a week in which on convalescence from illness the doctor advised that a few days' change in the country was necessary before resuming work. In neither case was the insured person aware of the rule, and consent of the societies was not obtained in advance. When the claims for benefit were put in they were declined on account of the breach of rule. We do not say that the societies were not justified in their action. It is, of course, quite possible that even if applications for permission to leave the parish had been made in advance they would have been declined, or that intimation would have been made that sickness benefit would not accrue from the date of leaving. This would, doubtless, have depended on circumstances such as the nature of the illness and the judgment of the society as to the necessity of the change of air in order to enable the member to resume work.

It does, however, seem to us to be arguable that the insistence on the rule that permission must be obtained before it is possible for the member to go away for a change—if that change is shown to be necessary—in order that he may retain his right to continue in receipt of benefit is contrary to the intention of the Act. The primary condition governing the right to sickness benefit, as laid down in the Act, is incapacity for work, and a change of air during convalescence is often required completely to restore the capacity for work. It should surely be sufficient evidence in support of a claim that the doctor had advised the necessity for the change, even though the formal consent—which may take some days to obtain—has not been secured beforehand.

Whether we are right in this contention or not one point at least emerges, namely, the desirability that all members of approved societies should become acquainted with the rules of the society to which they belong. Too much has been left to chance in regard to such matters, but a little trouble expended in the mastery of the rules of his society will not in the long run be wasted effort on the part of a member of any approved society.

**NOTE.—Questions and Correspondence on all doubtful points are invited, and should be addressed to the Editor, The Hospital, 28, 29 Southampton Street, Strand, London, W.C., and marked "Insurance."**

